# Dysregulated non-coding *telomerase RNA component* and associated exonuclease *XRN1* in leucocytes from women developing preeclampsia-possible link to enhanced senescence

**DOI:** 10.1038/s41598-021-99140-z

**Published:** 2021-10-05

**Authors:** Tove Lekva, Marie Cecilie Paasche Roland, Mette E. Estensen, Errol R. Norwitz, Tamara Tilburgs, Tore Henriksen, Jens Bollerslev, Kjersti R. Normann, Per Magnus, Ole Kristoffer Olstad, Pål Aukrust, Thor Ueland

**Affiliations:** 1 Research Institute of Internal Medicine, Oslo University Hospital, Rikshospitalet, Oslo, Norway; 2 Department of Obstetrics, Oslo University Hospital, Rikshospitalet, Oslo, Norway; 3 National Research Center for Women’s Health, Oslo University Hospital, Oslo, Norway; 4 Department of Cardiology, Oslo University Hospital, Rikshospitalet, Oslo, Norway; 5 Faculty of Medicine, University of Oslo, Oslo, Norway; 6 Newton-Wellesley Hospital, Boston, MA USA; 7 Division of Immunobiology, Center of Inflammation and Tolerance, Cincinnati, OH USA; 8 Department of Pediatrics, University of Cincinnati College of Medicine, Cincinnati, OH USA; 9 Section of Specialized Endocrinology, Department of Endocrinology, Oslo University Hospital, Rikshospitalet, Oslo, Norway; 10 Centre for Fertility and Health, Norwegian Institute of Public Health, Oslo, Norway; 11 The Blood Cell Research Group, Department of Medical Biochemistry, Oslo University Hospital, Ullevål, Oslo, Norway; 12 Section of Clinical Immunology and Infectious Diseases, Oslo University Hospital, Rikshospitalet, Oslo, Norway; 13 K.G. Jebsen Inflammatory Research Center, University of Oslo, Oslo, Norway; 14 K. G. Jebsen Thrombosis Research and Expertise Center, University of Tromsø, Tromsø, Norway

**Keywords:** Biomarkers, Cardiology, Medical research, Molecular medicine, Pathogenesis, Risk factors

## Abstract

Senescence in placenta/fetal membranes is a normal phenomenon linked to term parturition. However, excessive senescence which may be induced by telomere attrition, has been associated with preeclampsia (PE). We hypothesized that the telomerase complex in peripheral blood mononuclear cells (PBMC) and circulating telomere associated senescence markers would be dysregulated in women with PE. We measured *long non-coding* (*nc*) *RNA telomerase RNA component* (*TERC*) and RNAs involved in the maturation of *TERC* in PBMC, and the expression of *TERC* and *5′–3′ Exoribonuclease 1* (*XRN1*) in extracellular vesicles at 22–24 weeks, 36–38 weeks and, 5-year follow-up in controls and PE. We also measured telomere length at 22–24 weeks and 5-year follow-up. The circulating senescence markers cathelicidin antimicrobial peptide (CAMP), β-galactosidase, stathmin 1 (STMN1) and chitotriosidase/CHIT1 were measured at 14–16, 22–24, 36–38 weeks and at 5-year follow-up in the STORK study and before delivery and 6 months post-partum in the ACUTE PE study. We found decreased expression of *TERC* in PBMC early in pregnant women who subsequently developed PE. *XRN1* involved in the maturation of *TERC* was also reduced in pregnancy and 5-year follow-up. Further, we found that the senescence markers CAMP and β-galactosidase were increased in PE pregnancies, and CAMP remained higher at 5-year follow-up. β-galactosidase was associated with atherogenic lipid ratios during pregnancy and at 5-year follow-up, in PE particularly. This study suggests a potential involvement of dysfunctional telomerase biology in the pathophysiology of PE, which is not restricted to the placenta.

## Introduction

Pregnancy is a state of oxidative stress as the higher metabolic demand of the growing fetus results in increased reactive oxygen species production^1,2^. Oxidative stress is implicated in the pathophysiology of many reproductive complications including infertility, miscarriage and pre-eclampsia (PE), but the mechanisms of these complications are not fully elucidated. Cellular senescence, a condition in which a cell no longer has the ability to proliferate, can be activated by intrinsic and extrinsic factors like oxidative stress, inflammation, DNA damage and epigenetic stress. The increased senescence may be induced by telomere attrition (shortening), but also by long uncapped and dysfunctional telomeres, which have been associated with pregnancy complications, including PE^1,3^.

Telomere length is regulated by the enzyme telomerase that adds telomeric repeats to the ends of the chromosomes^4^. Telomerase is a ribonucleoprotein complex with a catalytic core composed of telomerase reverse transcriptase (TERT) and the *non-coding RNA* (*ncRNA*) *TERC*. Regulation of telomerase activity occurs through the control of *TERT* transcription and through a post-transcriptional maturation process of the 3′ end of *TERC*, which determines circulating levels of mature *TERC* and mature telomerase complex. *TERC* transcripts undergo a multistep process of maturation that includes cycles of adenylation and de-adenylation, which together control steady state circulating levels of *TERC*^4^. These mechanisms are crucial for the regulation of telomerase levels, and subsequent telomere length, and are disrupted in many disease states^4^. Shorter telomeres and decreased expression of TERT has been reported in trophoblasts from PE patients, and may reflect a process of accelerated telomere shortening during pregnancy due to increased stress including oxidative stress^5,6^. A progressive physiological senescence and aging of decidual cells and placental membranes may be important for the onset of labor at term, whereas premature aging related to telomere pathology may lead to PE^6^.

In age-related diseases, telomere shortening is accelerated and has become the proxy parameter for overall poor health. *TERC*^−/−^ mice with critically short telomeres develop excessive inflammation, oxidative stress, endothelial dysfunction, and hypertension^7,8^, all features that are typical in PE. Furthermore, shorter leukocyte telomere length is associated with a long-term risk of aging and cardiovascular disease (CVD)^9–11^. Circulating senescence markers of human aging and several diseases associated with telomere shortening have been identified^12^. A meta-analysis of 22 studies with > 6.4 million women including 258,000 women with preeclampsia adjusting for confounders identified a fourfold increase in future incident heart failure and a twofold increased risk in coronary heart disease, stroke and death due to CVD^13^. The risk of future CVD is highest associated with early-onset PE^14^.

In a pilot array from 22 to 24 weeks’ gestation in peripheral blood mononuclear cells (PBMC), including lymphocytes, monocytes, natural killer cells and dendritic cells, from women who subsequently developed PE and normotensive controls, we found lower ncRNA *TERC* in the PE women, suggesting dysfunctional telomerase biology. To further explore these novel data we measured 1) levels of ncRNA *TERC* and other mRNAs involved in the maturation of *TERC* in PBMC and extracellular vesicles at different time points during pregnancy, in term placenta, and at 5-year follow-up comparing PE and controls; 2) telomere length during pregnancy and at 5-year follow-up in PE and controls; 3) levels of the senescence markers cathelicidin antimicrobial peptide (CAMP), β-galactosidase, stathmin 1 (STMN1), and chitotriosidase/CHIT1 in two independent cohorts at different timepoints during pregnancy and at follow-up comparing PE and controls; and 4) evaluated if these senescence markers were associated with future risk of CVD as evaluated by atherogenic lipid ratios and arterial stiffness 5 years post-partum in women with PE and controls.

## Methods

**Figure 1:**
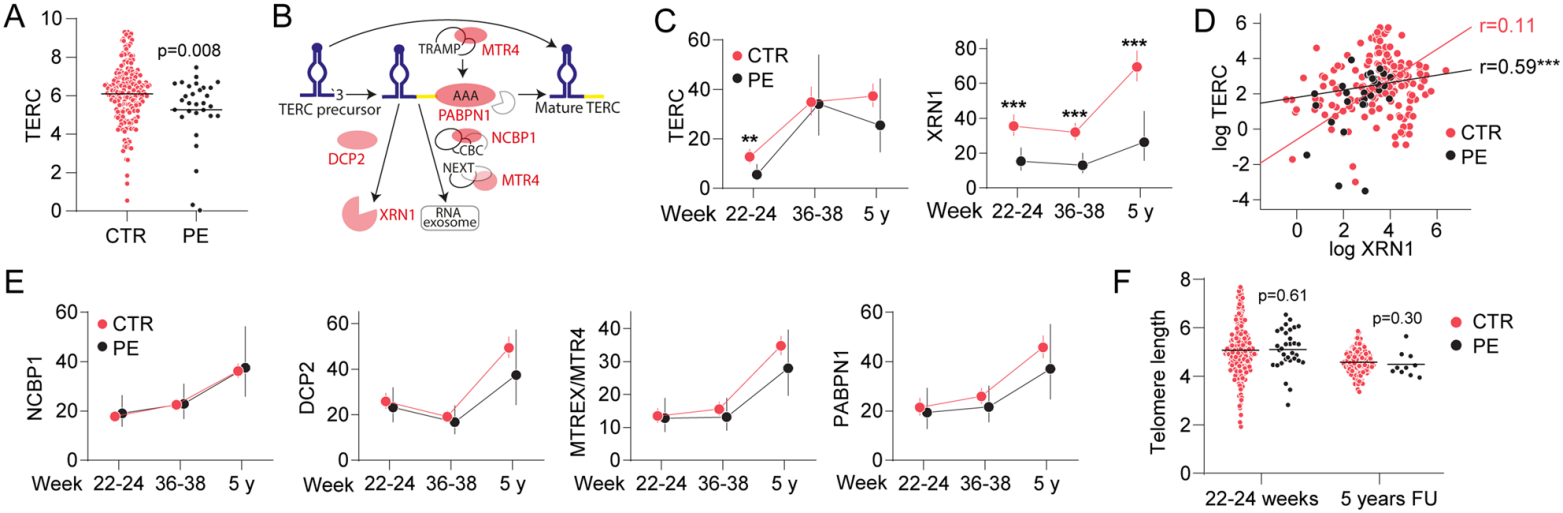
Expression levels of *TERC* and mRNAs of genes involved in the maturation of *TERC* and telomere length in PBMC (**A**) *TERC* expression from PBMC at 22–24 weeks in 182 controls and 38 PE (**B**) overview of the genes involved in the maturation of *TERC*, adapted from Roake et al.^4^ (Adobe Inc. (2019). Adobe Illustrator. Retrieved from https://adobe.com/products/illustrator) (**C** and **E**) longitudinal expression of *TERC* during pregnancy and follow-up and some of the genes involved in the maturation of *TERC* (**D**) correlation (Pearson) between *TERC* and *XRN1* within the control and PE group at 22–24 weeks gestation (**F**) telomere length in DNA from PBMC at 22–24 weeks and EDTA full blood at 5 years follow-up. **p < 0.01, ***p < 0.001.

The STORK study is a prospective longitudinal cohort study in which 1031 low-risk women of Scandinavian heritage with singleton pregnancies were followed throughout pregnancy and who gave birth at Oslo University Hospital Rikshospitalet between 2002 and 2008^15^. Exclusion criteria included pre-gestational diabetes and any severe chronic diseases (lung, cardiac, gastrointestinal or renal). Each pregnant woman had four study-related antenatal visits at 14–16, 22–24, 30–32, and 36–38 weeks. The follow-up study was performed 5-years after the index delivery in three hundred women^16^. In the current study women with gestational diabetes mellitus were excluded and we included only normotensive controls that were included in the follow-up study (215 controls, 38 PE in pregnancy and 10 PE at follow-up). In the ACUTE PE study there were 34 PE and 61 control subjects. Blood samples were collected at 25–38 weeks and at 6 months postpartum^17^. The cohorts are presented in Fig. 1, Supplemental File. Measurements of brachial arterial systolic and diastolic blood pressure (BP) were made with an automated oscillometric technique (Dinamap ProCare 300-Monitor, Criticon, GE Medical Systems). Systolic and diastolic BP were assessed as the mean of three recordings. Written informed consent was obtained from all study participants. All clinical investigations were conducted in accordance with the principles enshrined in the Declaration of Helsinki. The study was approved by the Regional Committee for Medical Research Ethics of Southern Norway in Oslo, Norway.

### Preeclampsia

PE was diagnosed by new-onset BP ≥ 140/90 mmHg and significant proteinuria (urinary total protein/creatinine ratio > 30 or + 1 on urine dipstick). In the STORK study, all cases (n = 38) were diagnosed after 34 weeks’ gestation (late-onset PE). The ACUTE PE study included cases diagnosed after 34 weeks (late-onset PE [n = 23]) and (early-onset PE [n = 11]).

### Collection, storage and RNA extraction of placental biopsies

As previously reported^18^, placental biopsies were collected after vaginal or cesarean delivery. Blocks of 2–4 cm were taken from the placental parenchyma, briefly washed in phosphate buffer saline, snap frozen in liquid nitrogen, and stored at − 80 °C until RNA isolation. Half of each biopsy was homogenized in TRIzol reagent (Invitrogen, Life Technologies) on ice with a tissue grinder (Sigma Aldrich, St. Louis, MO). Total RNA was extracted using TRIzol reagent (Invitrogen, Life Technologies) and purified with RNeasy microkit columns (Qiagen, Netherlands). Purity and concentration of isolated total RNA was measured using Nanodrop ND-1000 Spectrophotometer (Thermo Fisher Scientific Inc., USA) and RNA integrity number (RIN) was estimated using Agilent 2100 Bioanalyzer (Agilent Technologies, USA). Placental biopsies available for this study include 13 PE and 107 controls.

### Collection, storage and RNA extraction of PBMC

In the STORK study, PBMC was isolated from venous blood using BD Vacutainer CPT Tubes (BD, NJ) at weeks 22–24, 36–38, and at 5-year follow-up and stored at − 70 °C until extraction. RNA was extracted using Magnapure Isolation Kit and instrument (Roche Life Science, Penzberg, Germany) at weeks 22–24 and with Magmax isolation kit and instrument (Applied Biosystems, Carlsbad, CA) at weeks 36–38 and at follow-up, due to change in instruments at the laboratory over the years, as previously published^19^. RNA available from PBMC in this cohort included 189 controls/32 PE at 22–24 weeks, 194 controls/24 PE at 36–38 weeks and 213 controls/10 PE at 5-year follow-up.

### Microarray and data analysis

100 ng of total RNA from PBMC from 4 controls and 5 PE at week 22–24 was subjected to GeneChip HT One-Cycle cDNA Synthesis Kit and GeneChip HT IVT Labeling Kit, following the manufacturer’s protocol for whole genome gene expression analysis (Affymetrix, Santa Clara, CA). Labeled and fragmented single stranded cDNAs were hybridized to the GeneChip Human Gene 1.0 ST Arrays (28,869 transcripts) (Affymetrix). The arrays were washed and stained using FS-450 fluidics station (Affymetrix). Signal intensities were detected by Hewlett Packard Gene Array Scanner 3000 7G (Hewlett Packard, Palo Alto, CA). The scanned images were processed using Affymetrix GeneChip Command Console (AGCC). The CEL files were imported into Partek Genomics Suite software (Partek, Inc. MO). Robust microarray analysis (RMA) was applied for normalization. Differentially expressed genes between groups were identified using one-way ANOVA analysis. Cluster analysis were generated in Partek Genomics Suite. Further bioinformatics analysis was conducted on the significant genes to identify functional significance by means of Ingenuity Pathways Analysis (Ingenuity Systems, Redwood City, CA).

### Collection, storage and RNA extraction of extracellular vesicles

From the STORK study, 350 µl plasma from venous blood using BD Vacutainer CPT Tubes (BD, NJ) at weeks 22–24, 36–38, and at postpartum follow-up was used to isolate RNA from extracellular vesicles. The previous unthawed plasma stored at − 70 °C until extraction was thawed at room temperature centrifuged at 3000 g for 5 min and 300 µl was used further in the exoRNeasy plasma kit protocol (Qiagen)^20^. The spike-in control C. elegans miR-39 miRNA was added to the lysate after adding the Qiazol. We used 35 controls/35 PE at 22–24 weeks, 35 controls/27 PE at 36–38 weeks, and 35 controls/9 PE at 5 years follow-up for this analysis.

### Quantitative real-time polymerase chain reaction

Reverse transcription was performed using a High Capacity cDNA Archive Kit (Applied Biosystems, Foster City, CA) of RNA from PBMC and miScript II RT Kit Qiagen of RNA from extracellular vesicles. mRNA quantification was performed using SYBR Green PCR Fast Mix (Quantabio, Beverly, MA) for PBMC and miScript SYBR Green PCR Kit Qiagen for extracellular vesicles using the standard curve method on an ABI Prism 7900 (Applied Biosystems). Primers for *TERC* RT^2^ lncRNA qPCR Assay (LPH26581A) and Ce_miR-39_1 miScript Primer Assay was bought from Qiagen (Netherlands). Sequence specific intron spanning oligonucleotide primers were designed using the Primer Express software version 2.0 (Applied Biosystems) (Table 1, Supplemental File). Transcript expression levels were normalized to β-actin and GAPDH and expressed as relative mRNA levels in PBMC and geometric mean of β-actin, GAPDH, and miR-39 in extracellular vesicles.

**Table 1:** Maternal characteristics in controls and preeclamptic (PE) pregnancies. Data given as mean ± SD when normal distributed and median (25th, 75th) when skewed distributed. *BMI* body mass index, *MAP* mean arterial pressure, *FU* follow-up.

STORK study	Controls	PE	*p*-value
*N*	215	38	
Age (years)	32 ± 4	30 ± 4	0.001
Gestational age at delivery (week)	40 ± 1	39 ± 3	< 0.001
Multiparous n (%)	110 (47)	11 (29)	0.035
BMI (kg/m^2^) 14–16 week	23.5 (21.3, 25.4)	27.4 (23.1, 29.9)	< 0.001
BMI (kg/m^2^) 22–24 week	24.8 (22.5, 26.8)	29.0 (23.8, 32.1)	< 0.001
BMI (kg/m^2^) 30–32 week	26.1 (23.7, 28.3)	31.2 (26.0, 33.3)	< 0.001
BMI (kg/m^2^) 36–38 week	27.2 (24.8, 29.5)	32.2 (27.3, 34.8)	< 0.001
BMI (kg/m^2^) 5 years FU	22.6 (20.8, 24.5)	23.0 (20.1, 25.8)^a^	0.800
MAP (mmHg) 14–16 week	80 (75, 85)	85 (80, 90)	< 0.001
MAP (mmHg) 22–24 week	80 (73, 85)	83 (79, 93)	0.001
MAP (mmHg) 30–32 week	82 (77, 87)	87 (83, 95)	< 0.001
MAP (mmHg) 36–38 week	85 (80, 93)	100 (90, 108)	< 0.001
MAP (mmHg) 5 years FU	79 (73, 85)	81 (74, 84)^a^	0.888

ACUTE PE study	Controls	PE	*p*-value
*N*	61	34	
Age (years)	32 ± 5	32 ± 6	0.151
Gestational age (weeks)^b^	36 ± 0	35 ± 4	< 0.001
Gestational age at delivery (weeks)	40 ± 1	35 ± 4	< 0.001
Multiparous (%)	27 (42%)	13 (33%)	0.10
BMI (kg/m^2^)^b^	27.3 (24.6, 28.9)	30.1 (25.8, 34.2)	0.001
BMI (kg/m^2^) 6 months FU	22.8 (21.2, 25.2)	25.3 (22.1, 30.1)	0.010
MAP (mmHg)^b^	85 ± 8	116 ± 10	< 0.001
MAP (mmHg) 6 months FU	86 ± 8	98 ± 11	< 0.001

^a^N = 10 PE.

^b^At blood sampling.

### Biochemical analysis

Peripheral venous blood was drawn in the morning between 07:30 and 08:30 AM after an overnight fast, into tubes with citrate additives in the STORK study and EDTA additives in the ACUTE PE study, centrifuged for 25 min at 3000* g* at 4 °C, separated, and stored at − 80 C until analyzed. Fasting plasma levels of CAMP and STMN1 were measured in duplicate by enzyme immuno-assay with antibodies obtained from Mybiosource (San Diego, CA). β-galactosidase and chitotriosidase/CHIT1 were measured in duplicate by fluorescence. Briefly, 20 µl of citrate plasma and standards rhGLB1 (R&D Systems, Minneapolis, MN) diluted in 50 mM sodium citrate (pH 3.5) and 20 ul of 1.2 mM substrate (4-methylumbelliferyl-β-D-galactopyranoside (Sigma-Aldrich) was loaded into 384-well black plates. The plates were incubated in 37 °C for 1 h and the reaction stopped by 40 µl of 0.17 M glycine-carbonate buffer (pH 9.8) and the plates read by the fluorescent plate reader. For chitotriosidase/CHIT1 measurement, 5 µl citrate plasma and standards rhCHIT1 (R&D Systems) diluted in 10 mM MES assay buffer (pH 6) and 5 µl 200 µM substrate (4-methylbelliferyl β-D-N,N,N-triacetylchitotriose (Sigma-Aldrich) in assay buffer was loaded into 384-well black plates, mixed and incubated for 5 min protected from light and the plates read by the fluorescent plate reader. The β-galactosidase and chitotriosidase/CHIT1 activity was measured by excitation and emission wavelengths of 355 and 460 respectively. All 5 samples from one person were analyzed on the same plate. The variation (CV) for these assays in our hands were 17% for CAMP, 19% for STMN1, 7% for β-galactosidase, and 17% for chitotriosidase/CHIT1.

### Telomere length

DNA was extracted from the PBMC isolated from venous blood at weeks 22–24 (controls n = 186, PE n = 31) using Magnapure Isolation Kit and instrument (Roche Life Science) and using a salting out procedure^21^ of EDTA blood collected at the 5-year follow-up visit in the STORK study (controls n = 206, PE n = 10). Equal amounts of DNA (2 ng/μL) were used to measure telomere length by PCR using telomere‐specific primers relatively quantified to the single‐copy gene RPLP0 (Table 1, Supplemental File). All samples were run in duplicates. Quantification was performed using SYBR Green PCR Fast Mix (Quantabio, Beverly, MA) on an ABI Prism 7900 (Applied Biosystems).

### Statistical analysis

Statistical analyses were conducted using SPSS for Windows, version 21.0 (Chicago, IL). Data are expressed as mean ± SD when normally distributed and median (25^th^, 75^th^ percentile) when skewed. Comparison between women with PE and controls was performed using t-test or Mann–Whitney U depending on distribution, and Chi-square test for categorical variables. Associations were evaluated by Pearson correlation. We performed a mixed model analysis adjusting for age in the longitudinal PBMC, plasma and extracellular vesicles analysis. Two-tailed *p*-values < 0.05 were considered significant.

## Results

Table 1 shows the characteristic of the STORK study cohort^15,16^ and of the ACUTE PE study ^17^. In both populations, women with PE had significantly higher mean arterial pressure (MAP) and body mass index (BMI) during pregnancy as well as lower gestational age at delivery, while the women with PE were younger in the STORK study.

### *TERC* and mRNAs involved in the maturation of *TERC* from PBMC

The pilot array showed *TERC* as one of the top ten down-regulated transcripts (fold change − 1.8) from the ingenuity report in PBMC from PE women at 22–24 weeks (Table 2, Supplemental File). We then assessed *TERC* RNA in PBMC at 22–24 weeks and found a decreased expression in women with PE (Fig. 1A). We next investigated the temporal course and follow-up levels of *TERC* and some of the mRNAs involved in the maturation of *TERC* (*DCP2*, *XRN1*, *NCBP1*, *PABPN1*, *MTREX/MTR4*) (Fig. 1B). First, *TERC* increased from 22 to 24 weeks to term with similar levels at 5-year follow-up with no differences between groups at the two latter time-points (Fig. 1C). *XRN1* was markedly decreased at weeks 22–24, 36–38 and at 5-year follow-up in the women with PE (Fig. 1C). Further, *XRN1* and *TERC* were positively correlated at weeks 22–24 (*r* = 0.20, *p* = 0.003) with a stronger association in the PE group (Fig. 1D, r = 0.59, *p* = 0.001). Whereas *NCBP1* significantly increased, *DCP2* decreased during pregnancy (Fig. 1E). All other RNAs measured (*PABPN1*, *MTREX/MTR4*) were low during pregnancy and increased at the 5-year follow-up visit, except *TERC* which had similar levels at weeks 36–38.

**Table 2:** Associations between β-galactosidase, CAMP and lipids.

	14–16		36–38		5 years FU	
	Controls	PE	Controls	PE	Controls	PE
	*r* (*p*-value)	*r* (*p*-value)	*r* (*p*-value)	*r* (*p*-value)	*r* (*p*-value)	*r* (*p*-value)
**β-galactosidase**						
TG/HDL ratio	**0.19 (0.006)**	0.24 (0.148)	**0.21 (0.002)**	0.13 (0.488)	0.06 (0.356)	**0.82 (0.004)**
LDL/HDL ratio	**0.15 (0.025)**	**0.40 (0.013)**	**0.16 (0.018)**	**0.50 (0.006)**	− 0.12 (0.070)	0.59 (0.073)
**CAMP**						
TG/HDL ratio	0.05 (0.444)	0.18 (0.295)	− 0.01 (0.986)	0.20 (0.293)	0.03 (0.675)	− 0.09 (0.803)
LDL/HDL ratio	0.11 (0.099)	0.16 (0.357)	0.08 (0.237)	0.20 (0.297)	0.10 (0.168)	0.29 (0.425)

Significant associations in bold.

### *TERC* and *XRN1* expression were not differentially regulated between control and PE term placenta samples

**Figure 2:**
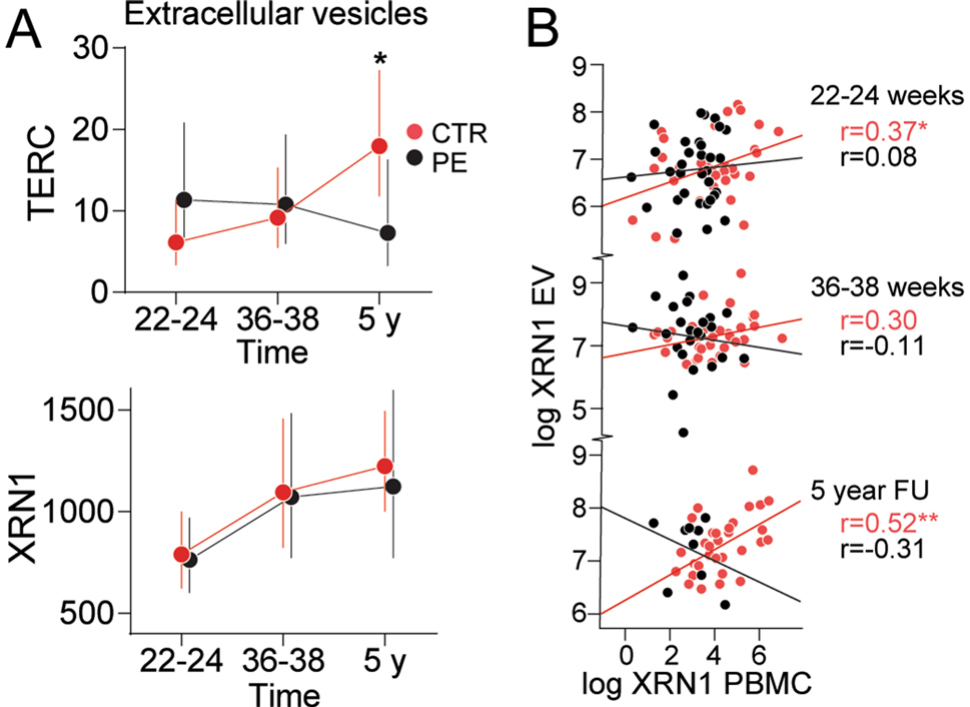
Expression of *TERC* and *XRN1* in extracellular vesicles and associations with expression in PBMC (**A**) Expression of *TERC* and *XRN1* in extracellular vesicles during pregnancy and 5 years follow-up in controls and PE. (**B**) Correlation plot (Pearson) between the expression of *TERC* and *XRN1* in extracellular vesicles and in PBMC at the different timepoints within the PE and control groups. *p < 0.05, **p < 0.01.

Although *TERC* and *XRN1* expression in PBMC was regulated and correlated in PE as compared with controls, this was not seen in term placentas (Fig. 2, Supplemental file). However, we did find an association between *TERC* levels in the term placenta and *TERC* in PBMC at week 36–38 (*r* = 0.21, *p* = 0.024) (data not shown).

### Telomere length were not differentially regulated between control and PE during pregnancy or at 5 years follow-up

We found no difference in the telomere length at 22–24 weeks or at 5-year follow-up comparing controls and women with PE (Fig. 1F). We also found an association between *XRN1* and telomere length (*r* = 0.15, *p* = 0.024) at 5-year follow-up in the whole cohort and in the PE group (*r* = 0.83, *p* = 0.005) (data not shown).

### *TERC* expression in extracellular vesicles from plasma was decreased in PE at 5 years postpartum

PBMC *TERC* levels increased during pregnancy and we wanted to explore its expression in extracellular vesicles, possibly reflecting feto-maternal crosstalk mechanisms. We found no increased expression of *TERC* and *XRN1* in circulating extracellular vesicles during pregnancy. However, *TERC* was significantly decreased in PE patients at 5-year follow-up (Fig. 2A). We found no difference in *XRN1* expression between controls and PE, but a significant correlation between *XRN1* in PBMC and *XRN1* in extracellular vesicles was found in controls at 22–24 weeks and at 5-year follow-up (Fig. 2B).

### The circulating senescence markers CAMP and β-galactosidase were increased in PE

**Figure 3:**
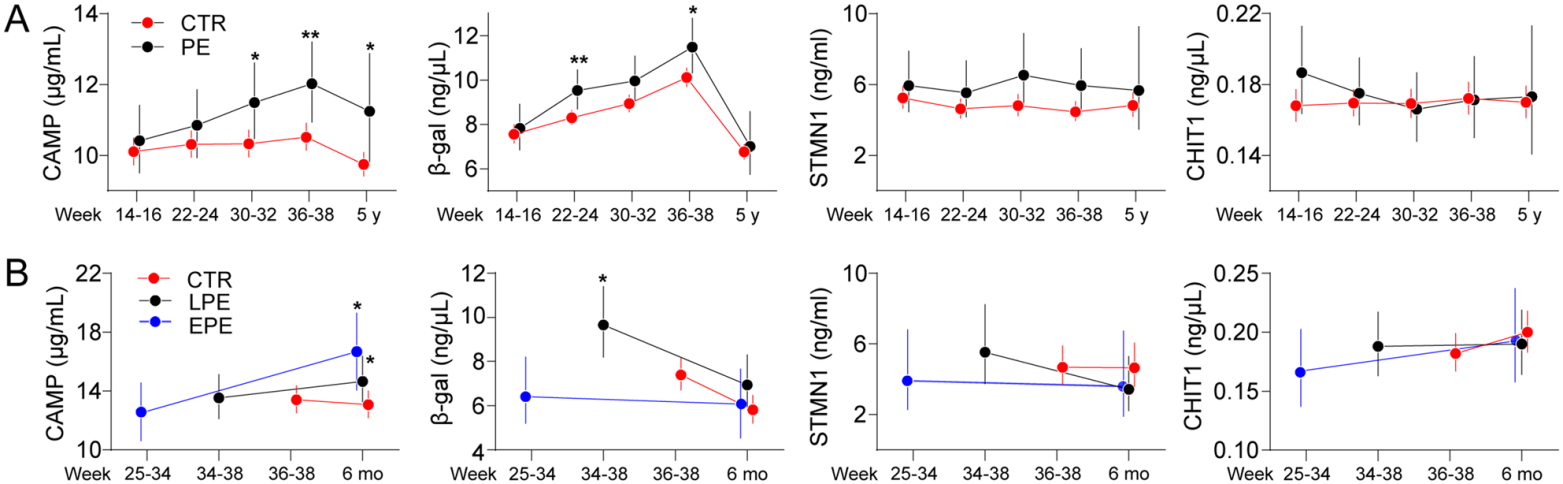
Temporal levels of circulating senescence markers during pregnancy and postpartum follow-up. (**A**) Levels of CAMP, β-galactosidase, STMN1 and chitotriosidase/chit1 during pregnancy and 5 years follow-up in the STORK cohort comparing controls and PE. (**B**) Levels of CAMP, β-galactosidase, STMN1 and chitotriosidase/chit1 during pregnancy and 6 months follow-up in the ACUTE PE cohort comparing controls and PE. *p < 0.05, **p < 0.001.

*TERC* expression and telomere length is related to senescence. As such we next investigated levels of circulating senescence markers CAMP, β-galactosidase, STMN1 and chitotriosidase/CHIT1 in the STORK and ACUTE PE study (Fig. 3). In the STORK study, we found β-galactosidase increased at 22–24 and 36–38 weeks, and CAMP increased at 30–32, 36–38 and at 5 years follow-up in women with PE compared to controls, after adjusting for age. CAMP and β-galactosidase increased significantly from 14 to 38 weeks during pregnancy in the whole cohort, while decreasing again at 5-year follow-up. Investigating the same markers in the ACUTE PE study we found CAMP increased at 6 months both in early- and late-onset PE, and β-galactosidase increased in late-onset PE during pregnancy compared to controls. We found no significant difference in the STMN1 and chitinase markers between controls and women with PE after adjusting for age or any significant changes during pregnancy or compared to follow-up. Thus, the senescence markers CAMP and β-galactosidase seems to be increased during PE pregnancies.

### CAMP and β-galactosidase were associated with atherogenic lipid ratios during pregnancy

We then investigated whether CAMP and β-galactosidase were associated with CV risk as reflected by the atherogenic lipid ratios TG/HDL-C and LDL/HDL-C^22^. In the control group in the STORK study, the TG/HDL-C ratio and LDL/HDL-C ratio were associated with β-galactosidase at week 14–16 and 36–38 (Table 2). In the PE group, the LDL/HDL-C ratio correlated with β-galactosidase during pregnancy and the TG/HDL-C ratio during postpartum follow-up. We found few associations between these markers at 5-year follow-up, although this may be due to smaller numbers of PE patients. We did not find any association between the senescence markers and arterial stiffness at 5-year follow-up in the STORK study or systemic arterial properties in the ACUTE PE study (data not shown).

### Few associations between BMI, telomere length, mRNAs involved in the maturation of *TERC* and senescence markers

Evaluated within PE and controls separately we observed no correlation between BMI and telomere length during pregnancy or at follow-up. We observed no correlations between BMI and *TERC* or *XRN1* RNA levels or with CAMP and β-galactosidase at any time-point during pregnancy or at follow-up with the exception of a correlation between BMI and β-galactosidase at week 14–16 (*r* = 0.19, *p* = 0.007) and at week 36–38 (*r* = 0.14, *p* = 0.044) in controls, but not in PE.

## Discussion

In the present study we found decreased expression of *TERC* in PBMC in pregnant women with PE. *XRN1* involved in the maturation of *TERC* was also low during pregnancy and at 5-year follow-up in patients with PE. Further, we found that the senescence markers CAMP and β-galactosidase were significantly elevated during pregnancy in women with PE, and CAMP remained elevated at 5-year follow-up. β-galactosidase was positively associated with atherogenic lipid ratios during pregnancy and at 5-year follow-up in women with PE.

Telomerase activity and cell senescence are altered during pregnancy and delivery since the placenta/fetal membranes are unique to this context, and there is evidence that the timing of these mechanisms may be temporally related to the timing of parturition^1,2,23^. There is also data to suggest that telomere-related cellular aging may be associated with PE, with premature activation of this pathway leading to placental villous telomere shortening, telomere aggregates, and trophoblast dysfunction in PE placentas^5,24–26^. Although we demonstrate changes in maternal leukocyte (PBMC) expression of *TERC* and senescence markers in PE, we were unable to detect any differences in telomere length in leukocytes or components related to telomere maturation between PE and controls in biopsies taken from placental parenchyma. Similar results were reported by previous studies comparing telomere length in circulating leukocytes from PE and normal pregnancies^27,28^ as well as leukocytes isolated from cord blood^29^. Furthermore, the similar maternal telomere length was not due to differences in age between the groups as we adjusted for age in the comparison and leukocytes were isolated at the same gestational age. As suggested by others, the lack of telomere shortening in blood leukocytes in PE suggests that these processes are probably restricted primarily to the placenta^24^. However, we found dysfunction in the markers of the telomerase complex in maternal leucocytes and propose that these processes are not restricted only to the placenta. For components related to telomere maturation between PE and controls in biopsies taken from placental parenchyma, it should be noted that the tissues examined were collected at delivery -often delivery at term-rather than at an earlier gestational age, and that specific cell lines within the placenta/fetal membranes were not separately examined. As the placenta secretes a range of biologically active substances, hormones, cells and extracellular vesicles into the maternal circulation, many of which may be taken up and affect cellular function within the maternal vasculature^30^, we speculate that the marked increase in *TERC* during pregnancy could reflect enhanced feto-maternal trafficking of extracellular vesicles. To this end, we did detect *TERC* in extracellular vesicles, but with a different temporal pattern which did not correlate with PBMC levels. However, RNA levels of *TERC* at 5-year follow-up were similar to term levels arguing against accumulation of placental derived *TERC* in PBMC during pregnancy.

A major finding in our study was the decreased levels of *TERC* and the RNA exonuclease *XRN1* as early as 22–24 weeks and at 5-year follow-up in PE patients, suggesting a possible underlying predisposition in such women that is unmasked during pregnancy, further supported by the strong positive correlation between *TERC* and *XRN1* mRNA levels in PE. *XRN1* controls the digestion and removal of ncRNA and is important for the maturation of *TERC*, thereby maintaining telomere length^31–33^. Similar to *TERC*, *XRN1* was detected in circulating extracellular vesicles and correlated with mRNA levels in PBMC in normotensive women but not in PE, suggesting that extracellular vesicles derived *XRN1* may contribute to the disrupted cell-to-cell communication seen with PE.

The secreted proteins, CAMP, STMN1, β-galactosidase and chitinase, were previously identified as senescence markers in telomere-dysfunctional mice (G4mTerc^−/−^)^12^. The increase in CAMP and β-galactosidase during pregnancy may reflect the progressive natural physiological senescence and aging of decidual cells and placenta/fetal membranes important for the onset of labor. During pregnancy, placental extravillous trophoblasts invade the pregnant decidua, losing their replicative potential and entering a senescent state characterized by high β-galactosidase activity. Moreover, in support of increased CAMP and β-galactosidase activity in PE pregnancies, prior studies have shown increase staining in trophoblast collected from women with both early- and late-onset PE^25,34^. These differences were less pronounced in the ACUTE PE study compared with the STORK study, which may be explained by the shorter gestational age, especially in regards to early-onset PE. While it is tempting to speculate that the increased circulating CAMP and β-galactosidase in PE women may be a consequence of the telomerase dysfunction, we were unable to detect any correlation with the decreased *TERC* and *XRN1* levels in PBMC. However, whereas cellular senescence suggests decreased proliferation, the cells that are present have a strongly pro-inflammatory phenotype. In relation to this, the release of CAMP is essential in innate immunity, with the ability to modulate both local innate and adaptive immune responses^35–39^. Thus, enhanced levels could reflect persistent inflammation and high amounts of ncRNA in the circulation of PE women.

Senescent cells often show a global change in their metabolism, including enhanced glycogen and lipid storage, induction of fatty acid synthesis, and increased secretion of inflammatory molecules. The increase in CAMP at 5-year follow-up in the PE women is especially interesting for investigating well-recognized long-term adverse events after PE^40^, although we observed no association with atherogenic lipid ratios. However, we found that circulating β-galactosidase activity, another marker of cellular senescence, correlated with TG/HDL and LDL/HDL ratios during pregnancy, with a stronger association in PE. This is consistent with prior studies that have shown an association between PE and dyslipidemia^41^. However, as β-galactosidase activity was normalized in PE at 5-year follow-up, the impact of this finding is unknown. Although we found no strong correlation between BMI and senescence markers or telomere length, there are reports that show accumulation of senescent cells in obesity^42,43^ and senescence is linked to CVD^44,45^. Our PE women had higher BMI during pregnancy and we cannot exclude that some effect of premature senescence in PE on CV risk could be mediated through interaction with adipose tissue mass.

A limitation of the study is the low numbers of PE women followed-up after 5-year in the STORK cohort and the different number of samples available in the experiments. Also, evaluation of specific cell lines within the placenta/fetal membranes could reveal regional differences in telomere homeostasis. Several factors including serum starvation, confluent culture, H_2_O_2_ treatment, age of cells and pH may change β-galactosidase activity^46^. However, our experiment on plasma samples were controlled for age and performed under controlled conditions with the identical reagents with similar pH at the same day. Still, our clinical cohorts are well described, with temporal samples during pregnancy and at 5-year follow-up.

In the present study, we found low levels of the long ncRNA *TERC* in PBMC coupled with low circulating *XRN1* levels in women with PE compared with normotensive controls. This may reflect dysfunctional telomerase activity in PE women. Although we did not show a significant overall decrease in telomere length in PBMC in women with PE, it is possible that such changes may occur later or may be localized to uterine tissues and the placenta/fetal membranes. The telomere-associated senescence markers CAMP and β-galactosidase were increased in PE during pregnancy and CAMP remained elevated at 6 months and 5 years after pregnancy.

## Supplementary Information

Supplementary Information 1.
