# Author Correction: Dysregulated non-coding *telomerase RNA component* and associated exonuclease *XRN1* in leucocytes from women developing preeclampsia-possible link to enhanced senescence

**DOI:** 10.1038/s41598-021-00917-z

**Published:** 2021-11-15

**Authors:** Tove Lekva, Marie Cecilie Paasche Roland, Mette E. Estensen, Errol R. Norwitz, Tamara Tilburgs, Tore Henriksen, Jens Bollerslev, Kjersti R. Normann, Per Magnus, Ole Kristoffer Olstad, Pål Aukrust, Thor Ueland

**Affiliations:** 1 Research Institute of Internal Medicine, Oslo University Hospital, Rikshospitalet, Oslo, Norway; 2 Department of Obstetrics, Oslo University Hospital, Rikshospitalet, Oslo, Norway; 3 National Research Center for Women’s Health, Oslo University Hospital, Oslo, Norway; 4 Department of Cardiology, Oslo University Hospital, Rikshospitalet, Oslo, Norway; 5 Faculty of Medicine, University of Oslo, Oslo, Norway; 6 Newton-Wellesley Hospital, Boston, MA USA; 7 Division of Immunobiology, Center of Inflammation and Tolerance, Cincinnati, OH USA; 8 Department of Pediatrics, University of Cincinnati College of Medicine, Cincinnati, OH USA; 9 Section of Specialized Endocrinology, Department of Endocrinology, Oslo University Hospital, Rikshospitalet, Oslo, Norway; 10 Centre for Fertility and Health, Norwegian Institute of Public Health, Oslo, Norway; 11 The Blood Cell Research Group, Department of Medical Biochemistry, Oslo University Hospital, Ullevål, Oslo, Norway; 12 Section of Clinical Immunology and Infectious Diseases, Oslo University Hospital, Rikshospitalet, Oslo, Norway; 13 K.G. Jebsen Inflammatory Research Center, University of Oslo, Oslo, Norway; 14 K. G. Jebsen Thrombosis Research and Expertise Center, University of Tromsø, Tromsø, Norway

Correction to: *Scientific Reports* 10.1038/s41598-021-99140-z, published online 05 October 2021

The original version of this Article contained errors in the spelling of the authors names Tove Lekva, Marie Cecilie Paasche Roland, Mette E. Estensen, Errol R. Norwitz, Tamara Tilburgs, Tore Henriksen, Jens Bollerslev, Kjersti R. Normann, Per Magnus, Ole Kristoffer Olstad, Pål Aukrust, Thor Ueland, which were incorrectly given as T. Lekva , M. C. P. Roland, M. E. Estensen , E. R. Norwitz, T. Tilburgs, T. Henriksen, J. Bollerslev, K. R. Normann, P. Magnus, O. K. Olstad, P. Aukrust, T. Ueland.

The original Article and accompanying Supplementary Information files have been corrected.

