# Supplementary material for: Dysregulated non-coding telomerase RNA component and associated exonuclease XRN1 in leucocytes from women developing preeclampsia-possible link to enhanced senescence
Source: Sci Rep. 2021 Oct 5;11:19735. doi: 10.1038/s41598-021-99140-z (PMC8492805; doi:10.1038/s41598-021-99140-z)
Supplement: Supplementary file 1 — Supplementary Information 1. [file 41598_2021_99140_MOESM1_ESM.doc]

Supplemental File

**Dysregulated non-coding *Telomerase RNA component* and associated exonuclease *XRN1* in leucocytes from women developing preeclampsia-possible link to enhanced senescence**

Tove Lekva, Marie Cecilie Paasche Roland, Mette E. Estensen, Errol R. Norwitz, Tamara Tilburgs, Tore Henriksen, Jens Bollerslev, Kjersti R. Normann, Per Magnus, Ole Kristoffer Olstad, Pål Aukrust, Thor Ueland


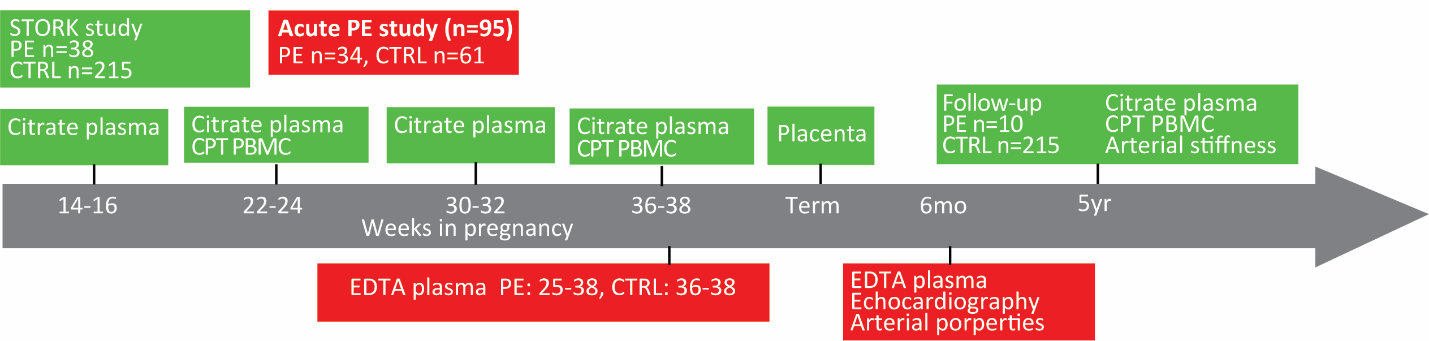


**Figure S1**. The timepoints for the preeclampsia patient (PE), controls (CTRL) and sample collections for the two cohorts used in this project.


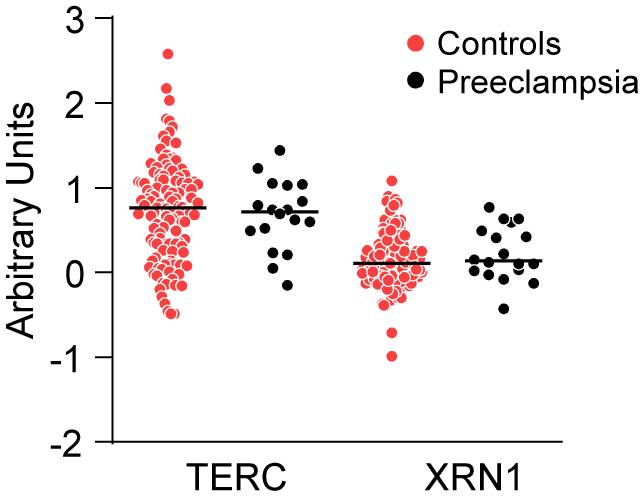


Figure S2. Gene expression of TERC and XRN1 in placenta term biopsies between controls and women with preeclampsia.

Table S1. Primer sequences used in PCR reactions.

| **Genes** | **GenBank** | **Forward (5´to 3´)** | **Reverse (5´to 3´)** |
| --- | --- | --- | --- |
| XRN1 | NM_001282859.2  NM_019001.5  NM_001282857.2 | TAGGTCAGCAAAGGAGGCAG | TGCCATGACTTGTCTGTGGA |
| NCBP1 | NM_001351505.2  NM_001351507.2  NM_002486.5 | AGAAGGCTTGGCTGGTGTTT | GGCGTGCAACTGTACAAAGA |
| PABPN1 | NM_004643.4  NM_001360551.3 | CATTGAGGACCCGGAGCTG | AATGGACATGATCACCGGGC |
| DCP2 | NM_152624.6 | GAGTCGAACCAAATTCCGCC | ATTCTGCTGCTTTGCTGGCT |
| MTREX | NM_015360.5 | CCACGAGTTGGAAAAGCTGC | AATGGCATACTCGGCGCATA |
| β-ACTIN | NM_001101.3 | AGGCACCAGGGCGTGAT | TCGTCCCAGTTGGTGACGAT |
| GAPDH | NM_001256799.1  NM_002046.4  NM_017008.3 | CCAAGGTCATCCATGACAACTT | AGGGGCCATCCACAGTCTT |
| Telomere |  | CGGTTTGTTTGGGTTTGGGTTTGGGTTTGGGTTTGGGTT | GGCTTGCCTTACCCTTACCCTTACCCTTACCCTTACCCT |
| RPLP0 |  | CAGATTGGCTACCCAACTGTT | GGAAGGTGTAATCCGTCTCCAC |

Table S2. Top diseases, functions, networks and molecules from the Ingenuity Pathway Analysis in the pilot array PBMC from women with preeclampsia at week 22-24

| **Top diseases and functions** | **p-value** | **Molecules** |
| --- | --- | --- |
| *Diseases and Disorders* | | |
| Connective Tissue Disorders | 3.84x10-^5^ | 26 |
| Hematological Disease | 3.84x10-^5^ | 13 |
| Organismal Injury and Abnormalities | 2.25x10-^4^ | 28 |
| Neurological Disease | 2.42x10-^4^ | 23 |
| Resipratory Disease | 3.04x10-^4^ | 10 |
| *Molecular and Cellular Functions* | | |
| Cell Death and Survival | 3.84x10-^5^ | 27 |
| Lipid Metabolism | 5.34x10-^5^ | 27 |
| Small Molecule Biochemistry | 5.34x10-^5^ | 32 |
| Molecular Transport | 5.78x10-^5^ | 27 |
| Cellular growth and Proliferation | 7.95x10-^5^ | 45 |
| *Physiological System Development and Function* | | |
| Hematological System Development and Function | 7.95x10-^5^ | 27 |
| Behavior | 2.25x10-^4^ | 2 |
| Hematopoiesis | 9.98x10-^4^ | 16 |
| Tissue Morphology | 1.05x10-^3^ | 32 |
| Embryonic Development | 1.09x10-^3^ | 17 |
| **Top Regulatory Effect Networks** | | |
| **Regulators** | **Diseases and Function** | **Consistency score** |
| IL1B | Differentiation of lymphocytes | 3.4 |
| TNF | Immune response of cells | -4.2 |
| TNF | Inflammation of body cavity | -4.9 |
| TNF | Quantity of antigen presenting cells | -5.4 |
| IL2 | Quantity of antigen presenting cells | -6.0 |
| **Top Networks** | | |
| **Associated Network Functions** | | **Score** |
| Lipid metabolism, small molecule biochemistry, cell signaling | | 30 |
| Cell-to-cell signaling and interaction, inflammatory response, hematological system development and function | | 26 |
| Connective tissue disorders, hematological disease, cellular assembly and organization | | 26 |
| Cellular function and maintenance, molecular transport, small molecule biochemistry | | 24 |
| Hereditary Disorder, organismal injury and abnormalities, renal and urological disease | | 24 |
| **Top Molecules** | | **Fold change** |
| *Up-regulated in preeclampsia* | |  |
| TMEM176A | | 3.3 |
| SDAD1 | | 2.5 |
| IL1B | | 2.1 |
| mir-181 | | 2.0 |
| mir-223 | | 2.0 |
| FOSB | | 1.9 |
| LY96 | | 1.9 |
| RAB7A | | 1.9 |
| RGS2 | | 1.9 |
| C12orf5 | | 1.9 |
| *Down-regulated in preeclampsia* | |  |
| LBHD1 | | -2.3 |
| RPPH1 | | -2.3 |
| HBB | | -2.2 |
| TERC | | -1.8 |
| PAX5 | | -1.7 |
| RPS20 | | -1.7 |
| CDKN2D | | -1.7 |
| R3HDM4 | | -1.7 |
| GLTSCR2 | | -1.7 |
| FAM90A27P | | -1.6 |
